# Analysis of Autozygosity Using Whole-Genome Sequence Data of Full-Sib Families in Pikeperch (*Sander lucioperca*)

**DOI:** 10.3389/fgene.2021.786934

**Published:** 2022-01-17

**Authors:** Lidia De los Ríos-Pérez, Tom Druet, Tom Goldammer, Dörte Wittenburg

**Affiliations:** ^1^ Institute of Genetics and Biometry, Research Institute for Farm Animal Biology (FBN), Dummerstorf, Germany; ^2^ Unit of Animal Genomics, GIGA-R & Faculty of Veterinary Medicine, University of Liège, Liège, Belgium; ^3^ Institute of Genome Biology, Research Institute for Farm Animal Biology (FBN), Dummerstorf, Germany; ^4^ Molecular Biology and Fish Genetics, Faculty of Agriculture and Environmental Sciences, University of Rostock, Rostock, Germany

**Keywords:** autozygosity, genomic inbreeding, FROH, pikeperch, selection, animal breeding

## Abstract

Pikeperch (*Sander lucioperca*) has emerged as a high value species to the aquaculture industry. However, its farming techniques are at an early stage and its production is often performed without a selective breeding program, potentially leading to high levels of inbreeding. In this study, we identified and characterized autozygosity based on genome-wide runs of homozygosity (ROH) on a sample of parental and offspring individuals, determined effective population size (*N*_*e*_), and assessed relatedness among parental individuals. A mean of 2,235 ± 526 and 1,841 ± 363 ROH segments per individual, resulting in a mean inbreeding coefficient of 0.33 ± 0.06 and 0.25 ± 0.06 were estimated for the progeny and parents, respectively. *N*_*e*_ was about 12 until four generations ago and at most 106 for 63 generations in the past, with varying genetic relatedness amongst the parents. This study shows the importance of genomic information when family relationships are unknown and the need of selective breeding programs for reproductive management decisions in the aquaculture industry.

## Introduction

Pikeperch (*Sander lucioperca*) is one of the five species of the genus *Sander* from the Percidae family. It is a fresh and brackish water fish with a native distribution in Eastern Europe and Western Asia, inhabiting the drainages basins of the Caspian, Baltic, Black, Aral, North, and Aegean Sea basins (Kottelat and Freyhof, 2007; Kestemont et al., 2015) and has been introduced to other countries from Europe, Asia, and North America. The origins of pikeperch production date back to the 19th century in Central and Eastern Europe, where pikeperch was farmed in small quantities with carp (*Cyprinus carpio*) in earthen ponds. In the early 20th century, the production of pikeperch as restocking material for open waters began, being produced as monoculture or polyculture with carp. In the second half of the 20th century, pikeperch production in extensive systems started to develop in Western Europe, and by the beginning of the 21st century, its production in recirculating aquaculture systems (RAS) was established (FAO, 2012). Although pikeperch farming techniques are at an early stage in Europe, its growing consumer demand has placed it as a candidate for aquaculture diversification, initiating over the last decades research projects to accelerate its production in intensive inland aquaculture systems (Zakęś et al., 2013; Kristan et al., 2018; Nguinkal et al., 2019; Thomas et al., 2020). Particularly in Germany, where pikeperch is also a native species, fish farmers and politics push to establish pikeperch as a new aquaculture species and thus to reduce the import from wild catches. However, to improve its production and economic profit, the use of domesticated stocks and the development of systematic and efficient breeding strategies are necessary.

The use of breeding programs implies the use of selective mating, where only some individuals will be chosen to transmit their genes to the next generation, which in the long term may lead to a change in the gene frequencies and a reduction of the effective population size (*N*_*e*_). This reduction implies an increased probability of mating between relatives. Populations undergoing selective mating experience some degree of inbreeding over time. The inbreeding coefficient (*F*) is the probability that two alleles at a randomly chosen locus are identical by descent (IBD), and it refers to the amount of inbreeding accumulated from a specific point in ancestry of the population (*e.g.*, Lewontin et al., 1968). Inbreeding is commonly associated with the reduction of mean phenotypic values of fitness traits, known as inbreeding depression (Falconer and Mackay, 1996; Lynch and Walsch, 1998). Thus, measuring individual inbreeding is critical in populations undergoing selection programs (Chavanne et al., 2016).

Prior to genomic information becoming available, pedigree-based relationships were used for estimating inbreeding. Pedigree inbreeding (*F*_PED_) is based on Mendelian sampling and does not consider concrete recombination events during meiosis. Furthermore, the assumption that the founder individuals are unrelated, parentage errors, and incomplete information lead to biased inbreeding estimates (Purfield et al., 2012; Forutan et al., 2018). Nowadays, genotyping technologies allow for genomic-based or combined approaches to more accurately estimate inbreeding (Zhang et al., 2015a). One genomic approach is through runs of homozygosity (ROH). ROH are continuous homozygous segments of the genome which can be identical by state or identical by descent. The latter are mostly interpreted as homozygous-by-descent (HBD) or autozygous segments, and can, for instance, be identified through the analysis of high-density single nucleotide polymorphism (SNP) panels (McQuillan et al., 2008; Ferenčaković et al., 2013b). ROH allow differentiating between recent and more ancient inbreeding. Long ROH segments indicate recent inbreeding, where recombination has had fewer opportunities to break up the ROH, whereas short segments trace back to more remote ancestors with a higher number of recombination events (Thompson, 2013). The proportion of the genome lying in HBD segments provides an estimate of individual inbreeding (Kardos et al., 2015), termed *F*_ROH_ below.

In the present study, we used an ultra-high density SNP panel of a pikeperch sample of two generations to identify and characterize autozygosity based on genome-wide ROH, to determine *N*_*e*_ from linkage disequilibrium in the parental generation, and to assess relatedness among parental individuals. Since, to the best of our knowledge, neither a genome-based nor pedigree-based breeding program has been established yet for pikeperch in Germany, hence we expected to observe an increased level of individual autozygosity and relatedness among individuals. The application of genomic tools helps discovering non-optimal breeding decisions in the past. Our results will contribute to setting the basis for the design of breeding strategies towards the improvement of the aquaculture production in pikeperch.

## Methods

### Data

We analyzed genotype data of pikeperch families initially produced for a linkage analysis (De los Ríos-Pérez et al., 2020). The production was based on F0 individuals without information about pedigree and former breeding practices. About 20 matings were performed. Out of the entire pool of progeny, about 2000 individuals were chosen according to positive mass selection (excluding the very early growing individuals). Following the same strategy, about 200 breeding candidates were selected from this resource, constituting the parent (F1) generation. Eventually, 18 individuals were selected on visual inspection, and 7 matings were performed with a male:female ratio of 2:1 and 1:1 (one male was used twice); one nest needed to be discarded. In tanks with a 2:1 male:female ratio, only one male was expected to fertilize the eggs. The 18 selected parents (11 males and 7 females) and 375 progeny were tissue sampled for DNA extraction and whole genome paired-end sequencing, followed by genotyping for SNP identification. Pedigree reconstruction was performed to identify the successful male in 2:1 matings and the progeny belonging to each mating. Further processing of the data yielded a final panel of 992,340 genome-wide SNPs from 11 parents (5 males and 6 females) and 363 progeny of 6 families. The number of progeny corresponding to each mating is shown in Table 1.

**TABLE 1:** Matings and number of individuals randomly sampled from a common tank.

Family	Sire Id	Dam Id	Number of progeny
1	1	2	28
2	3	4	89
3	5	6	3
4	7	8	223
5	9	10	14
6	9	11	6

Our analyses considered only markers within the 24 chromosomes of pikeperch, excluding those positioned in unplaced scaffolds, yielding a total of 992,313 SNPs on 2,709.64 centiMorgan (cM) or 896.05 Mega base pairs (Mb) on a sex-averaged map. Since the data came from an inbred population, no Hardy-Weinberg equilibrium or minor allele frequency filtering was performed.

### Runs of Homozygosity and Autozygosity

Runs of homozygosity analysis was performed with the RZooRoH package version 0.2.3 in R (Druet and Gautier, 2017; Bertrand et al., 2019). This package identifies HBD segments and estimates individual autozygosity. The model used by RZooRoH is a hidden Markov model (HMM) that partitions the genome-wide individual autozygosity into different HBD classes with pre-defined rates of ancestry change (*R*_*k*_) that are related to the age of inbreeding. In RZooRoH, the marker positions should ideally be provided in genetic distances in cM units. Alternatively, physical distances in base pairs (bp) can be used, assuming 1 Mb = 1 cM. In pikeperch, this assumption fails, as shown in the study performed by De los Ríos-Pérez et al. (2020). Therefore, we transformed the marker positions from Mb to cM by multiplying by 3.024 (= 2,709.64 cM/896.05 Mb). We analyzed progeny and parents separately, both based on the allele depth of the markers. To avoid bias due to the large difference of family sizes, only the allele frequencies of the 18 individuals used as putative parents were considered in the model parameters. We applied a “mixKR model” with 14 HBD classes with rate *R*_*k*_ = 2^k^ (i.e., 2^1^, 2^2^, 2^3^, … , 2^14^), related to the age of inbreeding, and one non-HBD class. Hence, HBD segments can have different lengths depending on HBD (age) class. The number of heterozygous SNPs allowed in a segment depends on a small probability justifying genotyping errors; as recommended we considered a small seqerror = 0.005. Consequently, too many heterozygous loci will cut a segment into shorter ROH. Eventually, total autozygosity was estimated for progeny and parents as accumulated autozygosity over all HBD classes, i.e.,

$$F_{ROH}=\sum_{k=1}^{14}F_{Rk}$$

where $F_{Rk}$ is the proportion of genome attributed to ROH segments in the *k*th HBD class.

### Effective Population Size

*N*_*e*_ was derived from linkage disequilibrium between SNP pairs in parents according to Santiago et al. (2020), as implemented in the software package GONE. This approach enables the estimation of temporal *N*_e_ and accounts for accumulated drift effects but it circumvents the restriction that *N*_e_ follows a linear trend back in time as, for instance, in Corbin et al. (2012). For this analysis, genotype data of the parents and progeny were phased with ShapeIT version 2 (Delaneau et al., 2013). In GONE, we neglected the selection of a mapping function because only recombination rates less than 0.05 (default setting) were taken into account.

Additionally, we performed nucleotide diversity analysis with HBD segments excluded similarly to “diversity outside ROH” used in Bosse et al. (2012), reflecting more ancient population size. Nucleotide diversity (π) was calculated in windows of 10 kilobases (kb) using VCFtools version 0.1.16 (Danecek et al., 2011) and averaged over the genome.

### Genetic Relatedness

Estimation of genetic relatedness between pairs of parental individuals was performed with ngsRelate version 2 (Hanghøj et al., 2019). This procedure utilizes identity-by-descent of SNP alleles and provides a scaled measure of relatedness in [0,1] (Hedrick et al., 2015).

## Results

### Runs of Homozygosity

A total of 811,440 ROH segments were identified in the progeny generation, with a mean of 2,235.37 ± 525.55 segments per individual and ranging from 741 to 3,279. The mean ROH length was 0.38 ± 1.59 cM, with the longest segment being identified on chromosome 15 with 75.78 cM and 25,207 SNPs. A total of 3,085 ROH segments were longer than 10 cM.

**FIGURE 1:**
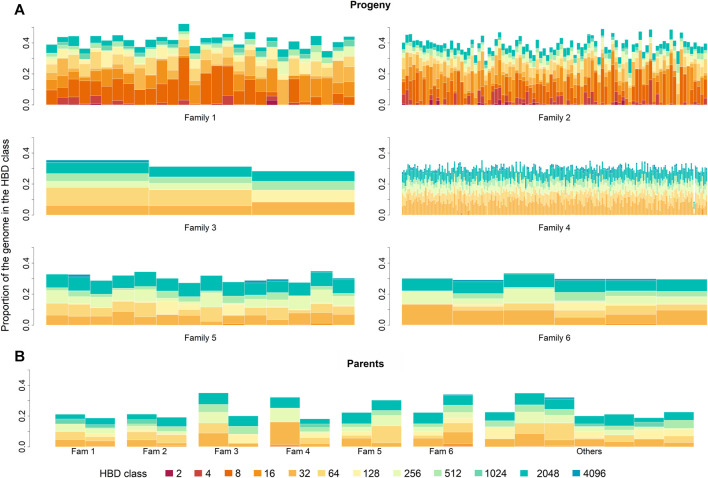
Partitioning of the genome in different homozygous-by-descent (HBD) classes for progeny **(A)** and parental **(B)** generation. Progeny generation is shown family-wise. Parental generation is separated by parents of each family. Family 5 and 6 built one paternal half-sib family. The height of the bar represents the proportion of the genome associated with the HBD class of the corresponding color.

In the parental generation, 33,146 ROH segments were identified with a mean of 1,841.44 ± 362.92 segments per individual, and ranging from 1,261 to 2,369. The mean ROH length was 0.34 ± 1.01 cM, with the longest segment identified in chromosome 17 with 25.31 cM and 10,996 SNPs. The ROH analysis for different age-related inbreeding classes showed that in both generations the highest number of segments corresponded to short segments (*R*_*k*_ = 2,048) from ancient inbreeding events 1,024 generations ago (≈*R*_*k*_/2) (Druet and Gautier, 2017; Bertrand et al., 2019). No segments were found for the oldest classes (i.e., *R*_*k*_ ≥ 8,192 and *R*_*k*_ ≥ 4,096 for the progeny and parental generations, respectively). The distribution of ROH over HBD classes in parents and progeny is summarized in Table 2. The partitioning of the genome in the different HBD classes is shown in Figure 1.

**TABLE 2:** Descriptive statistics of runs of homozygosity (ROH) and autozygosity contributions for the different age-related homozygous-by-descent (HBD) classes with pre-defined rates of ancestry change (*R*_*k*_) for progeny and parents. cM, centiMorgan; CUM, cumulative autozygosity.

Generation	HBD class (*R*_*k*_)	Age of inbreeding event (≈*R*_*k*_/2)	Number of ROH	No. of individuals with ≥1 ROH	Number of SNPs			Length (cM)			Proportion of genome in HBD class			
					Min	Max	Mean	Min	Max	Mean	Min	Max	Mean	CUM
Progeny	2	1	10	7	14,147	27,344	20,019	34.87	71.16	61.13	1.81 × 10^−19^	3.56 × 10^−2^	9.55 × 10^−4^	9.55 × 10^−4^
	4	2	135	33	2,396	27,344	13,910	7.87	75.78	42.79	5.12 × 10^−18^	1.61 × 10^−1^	6.87 × 10^−3^	7.82 × 10^−3^
	8	4	2,095	86	49	28,236	5,672	0.29	74.91	16.77	3.04 × 10^−15^	2.77 × 10^−1^	3.02 × 10^−2^	3.80 × 10^−2^
	16	8	2,971	69	33	16,642	2,852	0.38	45.81	8.18	7.69 × 10^−10^	2.52 × 10^−1^	2.45 × 10^−2^	6.25 × 10^−2^
	32	16	18,094	293	27	7,332	1,502	0.29	19.06	4.41	1.66 × 10^−6^	1.76 × 10^−1^	7.15 × 10^−2^	1.34 × 10^−1^
	64	32	24,427	248	9	3,869	757	0.23	9.69	2.07	1.08 × 10^−4^	1.49 × 10^−1^	5.42 × 10^−2^	1.88 × 10^−1^
	128	64	5,124	38	20	2,861	400	0.16	3.40	1.07	5.06 × 10^−11^	9.73 × 10^−2^	8.20 × 10^−3^	1.96 × 10^−1^
	256	128	78,885	305	9	1,754	224	0.08	1.91	0.58	1.33 × 10^−7^	9.30 × 10^−2^	4.04 × 10^−3^	2.37 × 10^−1^
	512	256	57,370	231	10	1,635	129	0.08	0.90	0.32	2.95 × 10^−6^	7.31 × 10^−2^	2.70 × 10^−2^	2.64 × 10^−1^
	1,024	512	7,319	28	1	657	79	1.00 × 10^−6^	0.48	0.18	1.03 × 10^−15^	4.21 × 10^−2^	2.82 × 10^−3^	2.67 × 10^−1^
	2,048	1,024	606,219	362	1	488	36	1.00 × 10^−6^	0.46	0.07	9.66 × 10^−3^	1.05 × 10^−1^	6.07 × 10^−2^	3.27 × 10^−1^
	4,096	2,048	8,791	35	1	155	17	1.00 × 10^−6^	0.04	0.01	9.13 × 10^−14^	3.86 × 10^−2^	6.58 × 10^−3^	3.34 × 10^−1^
Parents	2	1	0	0	0	0	0	0	0	0	8.38 × 10^−15^	4.26 × 10^−4^	6.42 × 10^−5^	6.42 × 10^−5^
	4	2	0	0	0	0	0	0	0	0	1.18 × 10^−15^	1.14 × 10^−3^	1.61 × 10^−4^	2.25 × 10^−4^
	8	4	0	0	0	0	0	0	0	0	2.80 × 10^−14^	3.49 × 10^−3^	5.08 × 10^−4^	7.34 × 10^−4^
	16	8	3	3	4,977	10,186	7,041	17.09	24.14	21.24	9.20 × 10^−8^	1.34 × 10^−2^	2.72 × 10^−3^	3.45 × 10^−3^
	32	16	441	18	35	10,996	2,044	0.76	25.31	6.41	1.78 × 10^−2^	1.48 × 10^−1^	5.03 × 10^−2^	5.38 × 10^−2^
	64	32	953	12	16	3,821	888	0.42	9.48	2.60	5.46 × 10^−23^	1.10 × 10^−1^	4.18 × 10^−2^	9.59 × 10^−2^
	128	64	818	8	24	1,931	492	0.20	4.01	1.35	2.82 × 10^−10^	7.81 × 10^−2^	2.32 × 10^−2^	1.19 × 10^−1^
	256	128	2,660	13	11	1,331	251	0.12	1.88	0.66	6.95 × 10^−5^	8.49 × 10^−2^	3.52 × 10^−2^	1.54 × 10^−1^
	512	256	3,060	12	13	932	137	0.05	0.80	0.35	2.39 × 10^−8^	5.25 × 10^−2^	2.69 × 10^−2^	1.81 × 10^−1^
	1,024	512	1,227	6	12	324	88	0.02	0.40	0.21	2.99 × 10^−11^	3.22 × 10^−2^	8.40 × 10^−3^	1.89 × 10^−1^
	2,048	1,024	23,984	18	2	466	39	0.00	0.43	0.07	2.87 × 10^−2^	7.58 × 10^−2^	5.69 × 10^−2^	2.46 × 10^−1^
	4,096	2,048	0	0	0	0	0	0	0	0	8.48 × 10^−37^	1.05 × 10^−2^	9.86 × 10^−4^	2.47 × 10^−1^

**FIGURE 2:**
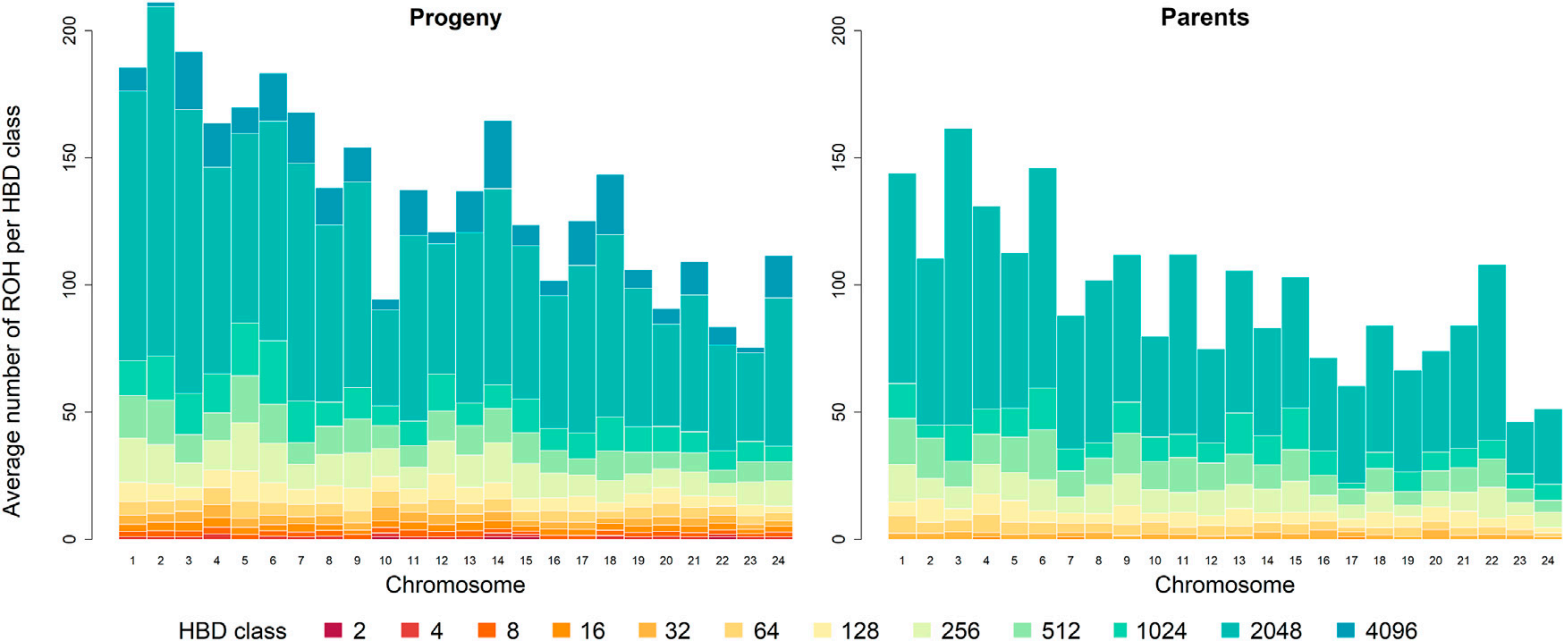
Distribution of the average number of runs of homozygosity (ROH) per individual for the different homozygous-by-descent (HBD) classes per chromosome in the progeny and parental generation. The height of the bar represents the average number of ROH segments associated with the different HBD classes of the corresponding color. Chromosomes are ordered by physical length, longest chromosome at first.

The chromosome-wise analysis of the average number of ROH per individual showed a dependence of ROH segments assigned to the different HBD classes on chromosome length in both generations, see Figure 2. As expected, the shorter a chromosome was, the less ROH were observed.

### Autozygosity

**FIGURE 3:**
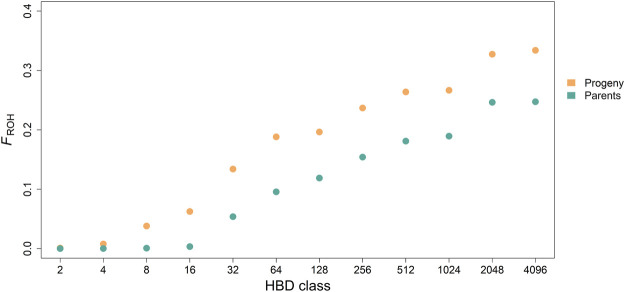
Accumulated average inbreeding coefficients (*F*_ROH_) through the different homozygous-by-descent (HBD) classes for progeny and parental generation.

Mean of estimated *F*_ROH_ was 0.33 ± 0.06 (0.25 ± 0.06) with a range from 0.09 to 0.52 (0.18–0.35) for the progeny (parent) generation. Mean of accumulated inbreeding coefficients over HBD classes for progeny and parental generations are shown in Figure 3. HBD classes *R*_*k*_ = 8 to 64 contributed most to total autozygosity in progeny and *R*_*k*_ = 32 to 512 in parents (also see Table 2). We considered HBD classes $R_{k}\leq 4$ as “recent” inbreeding. *F*_*Rk*_≤_4_ ranged from 0 to 0.16 with a mean of 7.82 × 10^−3^ ± 2.15 × 10^−2^ for the progeny generation. With progeny of families one and two excluded, recent inbreeding ranged only from 0 to 0.10. The increased occurrence of ROH segments in the most recent HBD classes for the progeny of families one and two suggest that the parents of each family were highly related (Figure 1). The parents had a mean *F*_*Rk* ≤ 4_ of 2.25 × 10^−4^ ± 4.59 × 10^−4^ ranging from 0 to 1.57 × 10^−3^. For parents, we found that inbreeding was relatively old: *F*_ROH_ was about 0.15 with respect to 128 generations in the past but moderately low at 0.05 if 16 generations were considered. However, since the parent generation is rather small, this outcome may be influenced by sampling variation and shall not be overvalued.

The progeny with the lowest *F*_ROH_ = 0.09 belonged to family 4 which had the largest sample size. With the single outlier excluded, the average inbreeding of its siblings was 0.34 ± 0.05, ranging between 0.25 and 0.52. No concrete reason was found to explain the difference in inbreeding coefficients between the outlier and its siblings.

### Effective Population Size

**FIGURE 4:**
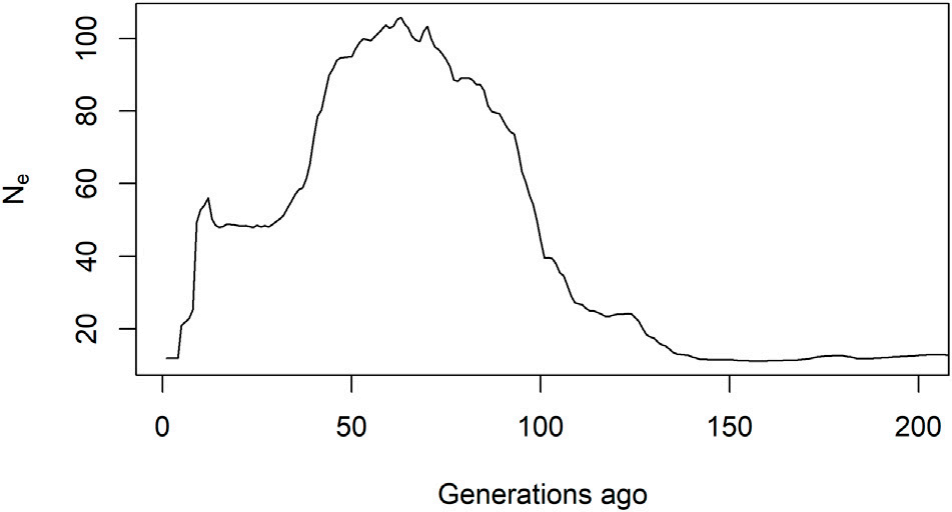
Estimates of historical *N*_*e*_.

Estimates of historical *N*_*e*_ are shown in Figure 4. Until four generations in the past, estimated *N*_*e*_ was extremely low at about 12 and increased steadily towards *N*_e_ = 106 for 63 generations ago. For more than 150 generations ago, *N*_e_ stabilizes at about 12. The particularly low *N*_*e*_ for the recent history agreed with our expectation on reproduction practices discarding relatedness.

Nucleotide diversity was on average 2.44 × 10^−4^ ± 2.13 × 10^−4^ per 10 kb window among all individuals.

### Genetic Relatedness

**FIGURE 5:**
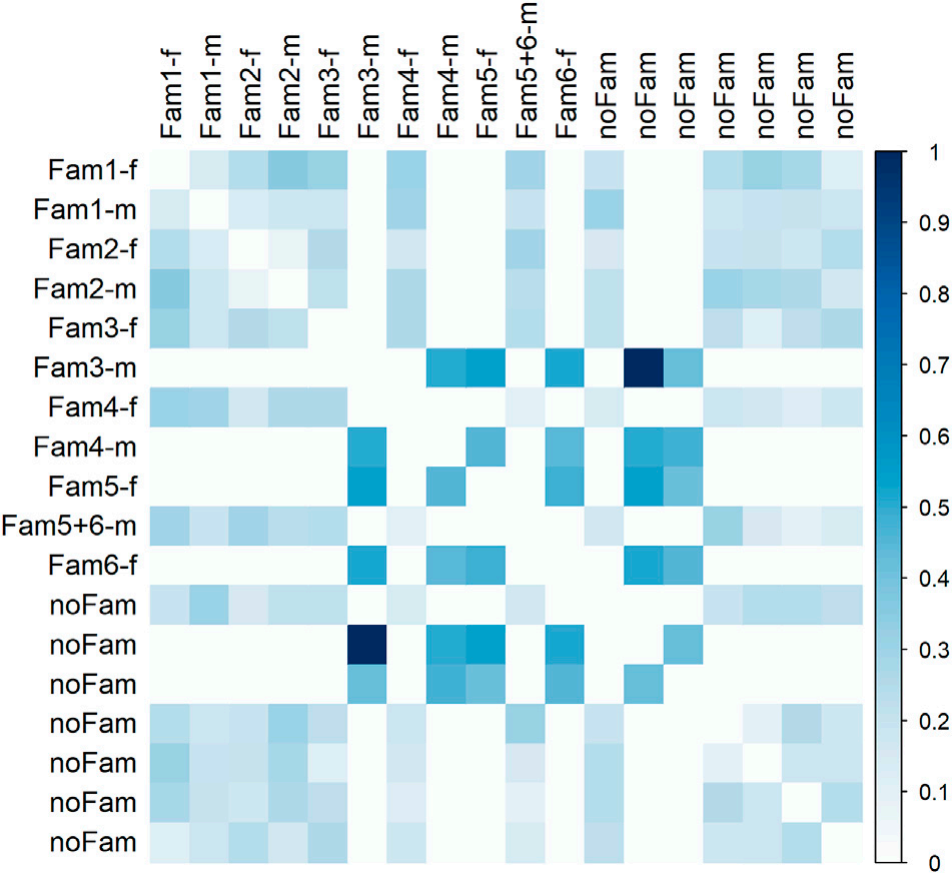
Relatedness matrix for the parent population. Column and row names refer to family assignment. In case of “noFam,” an individual was not used for mating; extension -f indicates females and -m males.

Most parental individuals were almost unrelated with regard to genotype data (Figure 5). A suspiciously high coefficient of relatedness was observed for two individuals, one of them was father of family three, with no obvious explanation. A group of four individuals, being parents of the progeny generation, revealed medium relatedness of about 0.5 among each other indicating common ancestor(s). Against our assumption from autozygosity analysis, a closer relationship between parents within family one and two was not confirmed; relatedness was 0.14 and 0.08, respectively.

## Discussion

We assessed genome-wide autozygosity in parents and progeny of a pikeperch population based on an ultra-high density SNPs panel. Our study represents the first investigation of inbreeding based on ROH in this species. Considering progeny and parents, our results showed that the majority of autozygosity is associated with ancestors over 16 generations ago (*R*_*k*_ ≥ 32). According to pikeperch history, the origins of its production dates back to the 19th century (FAO, 2012). Assuming a generation interval of 3–5 years, pikeperch production origins took place approximately 43–73 generations ago.

Genome-wide ROH analysis has been performed in multiple livestock species, such as cattle (Ferenčaković et al., 2013a; Peripolli et al., 2020), chicken (Dementieva et al., 2020), and pig (Bosse et al., 2015; Shi et al., 2020). However, only few studies are available on aquaculture species. ROH reported in rainbow trout (*Oncorhynchus mykiss*) showed a moderate level of autozygosity in several lines, with *F*_ROH_ ranging from 10.0% in an unselected experimental line to 19.5% in a commercial line (D’Ambrosio et al., 2019). In turbot (*Scophthalmus maximus*), large differences were found in ROH distribution between wild and domestic populations, with wild populations showing only short length segments (<2 Mb) (Aramburu et al., 2020). In coho salmon (*Oncorhynchus kisutch*), an analysis of ROH patterns between two pure lines and one admixed line showed a larger number and greater mean length of ROH in the pure lines (Yoshida et al., 2020). More precisely, mean ± SD *F*_ROH_ for two pure lines and one admixed line were of 14.2 ± 3.8%, 15.2 ± 4.4% and 0.4 ± 0.3%, respectively. As the specification of length of ROH varied among literature, a thorough comparison between studies is actually not possible. Nevertheless, in our study, *F*_ROH_ estimates for the progeny generation were found to range between 25 and 52%, which is considerably higher than the *F*_ROH_ estimates observed in aquaculture species undergoing a selection program. Unlike SNP array data used in previous studies, sequence data can also capture ancient inbreeding (through small HBD segments), consequently leading to higher and more precise estimates of *F*_ROH_ (Zhang et al., 2015a). Restricted to HBD classes with *R*_*k*_ ≤ 128 (mean segment length was ≥1 cM comparable to array data), mean autozygosity was then 19% among progeny. Evaluations and comparisons between ROH (in terms of IBS) and HBD methods showed that at high marker density, the estimated inbreeding coefficients are highly correlated and that the longest ROH are captured by both methods (Druet and Gautier, 2017; Solé et al., 2017; Alemu et al., 2021). The main difference is related to the capacity of the methods to measure autozygosity associated with the shortest HBD segments which capture background LD.

The observed nucleotide diversity was very low compared to mammals, such as cattle (Zhang et al., 2015b) or pigs (Bosse et al., 2012). Such low values have also been observed in other fish species, for instance, salmon (Kijas et al., 2018) and catfish (Jensen et al., 2021). Based on the relationship π = 4 *N*_*e*_ν with a mutation rate ν per sequence (Nei and Takahata, 1993), this outcome also strengthens the indication that past *N*_*e*_ size must have been rather small in pikeperch.

Estimates of *N*_*e*_ for recent generations (≤4 generations ago) were found well below the minimal recommended value of 50 individuals which are necessary to avoid inbreeding depression in the short term (FAO, 1998), and also lower than those estimated for other aquaculture species (e.g., Saura et al., 2021). In rainbow trout, *N*_*e*_ estimates in four commercial lines mainly selected for growth ranged from 37 to 48 for one generation in the past (D’Ambrosio et al., 2019). In a study performed in channel catfish (*Ictalurus punctatus*), *N*_*e*_ was calculated based on mean linkage disequilibrium between adjacent markers and on inbreeding *F*_PED_, where both methods gave similar results, i.e., 27 and 28, respectively (Garcia et al., 2018). A study performed in Atlantic salmon (*Salmo salar*) showed *N*_*e*_ estimates ranging from 15 to 72 few generations ago of three populations under study (Barria et al., 2018). Comparison between our results and those obtained in other aquaculture species following breeding programs makes doubtlessly clear that pedigree information and inbreeding levels should be closely monitored. Only then, breeders can ensure sufficient genetic diversity so that a population can adapt to future breeding goals and the accumulation of detrimental effects associated with inbreeding is avoided.

The results presented were based on RZooRoH’s mixKR model with parameters *K* = 15 and *R* = 2. We additionally evaluated a variety of model parameters to identify a statistically “optimal” number of HBD classes. To this end, models with *K* = 2 to *K* = 18 and *R* = 2 to *R* = 10 were compared using the Bayesian information criterion on individual likelihoods (Druet and Gautier, 2017). With the optimal parameter setting *K* = 5 and *R* = 7, estimates of total autozygosity were almost unaffected (Supplementary Table S1). We decided to present results allowing for a finer retrospect on demographical events.

### Implications of the Study

In the aquaculture industry, the large number of progeny per female might suggest the need of keeping only few broodstock to satisfy production requirements. Inadequate numbers of broodstock lead to a population susceptible to inbreeding. In the present study, the data was obtained from an aquaculture facility without a breeding program or an animal record of ancestry. This is reflected in the high levels of inbreeding obtained in the population, making evident the importance of knowing the genetic diversity and kinship relationships for reproductive management decisions. Therefore, the implementation of a breeding program is highly recommended to increase the genetic values of the population while controlling inbreeding. Additional measures might be required, such as importing pikeperch from other populations that contribute additional genetic variation. Furthermore, the integration of genomic tools in the design of a breeding program, such as SNP panels for parentage assignment and estimation of breeding values, will allow to better estimate genetic parameters, particularly inbreeding, in a population (Dekkers, 2012; Yáñez et al., 2015).

## Data Availability

Publicly available datasets were analyzed in this study. This data can be found here: The raw sequencing data are available at the NCBI Sequence Read Archive (SRA) under Accession Number PRJNA626522. The genome assembly of Sander lucioperca (SLUC_FBN_1.2) is available at the NCBI GenBank under the Accession Number GCA_008315115.2.
