# Supplementary material for: Analysis of Autozygosity Using Whole-Genome Sequence Data of Full-Sib Families in Pikeperch (Sander lucioperca)
Source: Front Genet. 2022 Jan 17;12:786934. doi: 10.3389/fgene.2021.786934 (PMC8801746; doi:10.3389/fgene.2021.786934)
Supplement: Supplementary file 1 [file Table1.pdf]

Supplementary Material

Table S1. Descriptive statistics of runs of homozygosity (ROH) and autozygosity contributions for the different age-related homozygous-by-descent (HBD) classes with pre-defined rates of ancestry change (R_k) for progeny and parents. cM, centiMorgan; CUM, cumulative autozygosity.

**Generation**	**HBD class** (R_k)	**Age of inbreeding event** ($\approx R_k / 2$)	**Number of ROH**	**No. of individuals with ≥1 ROH**	**Number of SNPs**			**Length (cM)**			**Proportion of genome in HBD class**			
					**Min**	**Max**	**Mean**	**Min**	**Max**	**Mean**	**Min**	**Max**	**Mean**	**CUM**
Progeny	7	3.5	3,842	160	384	28,236	5,450	1.33	75.78	23.00	8.97×10^{-7}	3.19×10^{-1}	5.86×10^{-2}	5.86×10^{-2}
	49	24.5	47,464	363	9	6,746	1,040	0.29	17.05	16.05	2.02×10^{-2}	1.89×10^{-1}	1.35×10^{-1}	1.94×10^{-1}
	343	171.5	180,092	363	8	1,650	157	0.05	1.43	5.11	3.00×10^{-2}	1.29×10^{-1}	7.45×10^{-2}	2.68×10^{-1}
	2,401	1,200.5	607,696	363	1	484	33	1.00×10^{-6}	0.73	1.54	2.55×10^{-2}	1.03×10^{-1}	6.56×10^{-2}	3.34×10^{-1}
	16,807	8,403.5	0	0	0	0	0	0	0	0	7.16×10^{-9}	1.65×10^{-5}	1.10×10^{-6}	3.34×10^{-1}
Parents	7	3.5	30	7	2,038	10,996	4,454	5.21	25.31	15.29	4.36×10^{-6}	6.27×10^{-1}	9.45×10^{-3}	9.45×10^{-3}
	49	24.5	1,905	18	45	6,341	1,031	0.45	17.25	3.07	5.38×10^{-2}	1.57×10^{-1}	1.07×10^{-1}	1.16×10^{-1}
	343	171.5	9,914	18	10	1,059	150	0.05	1.43	0.38	6.03×10^{-2}	1.17×10^{-1}	7.96×10^{-2}	1.96×10^{-1}
	2,401	1,200.5	21,488	18	3	329	35	1.10×10^{-4}	0.17	0.06	3.42×10^{-2}	6.77×10^{-1}	5.19×10^{-2}	2.48×10^{-1}
	16,807	8,403.5	0	0	0	0	0	0	0	0	3.52×10^{-10}	1.50×10^{-5}	1.60×10^{-6}	2.48×10^{-1}
